# Selective Adsorption of Fluorine Contaminants from Spiked Wastewater via a Novel Fe^III^–Ce^IV^-Based Layered Hydroxide Composite and Mechanism Analysis of Colloids and Surfaces

**DOI:** 10.3390/ma18112665

**Published:** 2025-06-05

**Authors:** Jing Du, Yanyan Zhao, Tao Huang, Hui Li, Jia He

**Affiliations:** 1 School of Materials Engineering, Changshu Institute of Technology, Suzhou 215500, China; 201700037@szut.edu.cn; 2 Changsha Institute of Mining Research Co., Ltd., Changsha 410012, China; 3 Huai’an Water Conservancy Survey and Design Institute Co., Ltd., Huai’an 223001, China; 4 Changshu Pufa Second Thermal Energy Co., Ltd., Changshu 215500, China; hejia_111@163.com

**Keywords:** adsorption mechanism, column-mode optimization, fluorine contaminant, Fe^III^–Ce^IV^-based layered hydroxide composite, selective removal

## Abstract

Excessive intake of fluorine (F) over time can lead to acute or chronic fluorosis. In this study, a novel Fe^III^–Ce^IV^-based layered hydroxide composite (DD-LHC) was synthesized and applied in both batch and column modes to develop new adsorbent materials and to obtain efficient removal of fluorine (F) anions from wastewater. DD-LHC achieved better adsorption results and material stability compared to green rusts (GR, Fe^II^–Fe^III^ hydroxide). The maximum adsorption capacity of DD-LHC for F^−^ was 44.68 mmol·g^−1^, obtained at an initial pH of 5 and initial concentration of 80 mM. The substitution of Ce^IV^ for Fe^II^ in the intercalated layered structure of GR potentially changed the reaction pathways for F^−^ removal, which are typically dominant in the layered double hydroxides (LDHs) of Fe^II^–Fe^III^. The molecular structure of layered hydroxides combined with the three-dimensional (3D) metal frame of Fe-O-Ce was integrated into DD-LHC, resulting in nanoscale particle morphologies distinct from those of GR. The pseudo-first-order kinetic model effectively described the whole adsorption process of DD-LHC for F^−^. DD-LHC exhibited notable selectivity for F^−^ across a wide pH range. The removal process of F^−^ by DD-LHC was dominated by Ce–F coordination bonds, with additional influences from auxiliary pathways to different extents.

## 1. Introduction

Fluorine (F) is the 13th most abundant element in the Earth’s crust. The distribution of the outer electron shell of F is 1s^2^2s^2^2p^5^(2p^0^_3/2_), giving F the highest electronegativity and making it the most reactive non-metal among all the elements in the periodic table. The element of F forms compounds with almost all other elements and exists in the form of compounds in nature. The F species stored in ores, bedrock, and sediments can be arbitrarily released into the soil and groundwater through uncontrolled human exploitation [1]. A large amount of F-containing wastewater is released into the environment from chemical production processes such as aluminum smelting, steel production, phosphate fertilizer, organic fluorine pesticides, glass processing, ceramics, semiconductors, pharmaceuticals, etc. [2,3,4]. F is an essential trace element for the human body, playing a key role in the formation of teeth and bones. However, excessive intake of F within a short or long period can lead to acute and chronic fluorosis [5,6]. Therefore, it is of great significance to remove F contaminants from water. F mainly exists in the form of fluorinion in drinking water. The recommended maximum concentration of F is limited to 1.0 mg·L^−1^ in drinking water according to the World Health Organization (WHO).

Several techniques have been adopted to remove F species from wastewater, such as electrocoagulation, membrane filtering, ion exchange, adsorption, chemical precipitation, etc. [4,7,8,9,10,11]. Adsorption treatment has showed the most promise among these techniques due to its intrinsic advantages [12,13,14]. Some inorganic adsorbents, natural polymers, and biomass materials have been employed for the removal of fluorinion [11,12,13,14,15,16,17,18]. Alumina-/aluminum- and magnesia-/magnesium-based materials and their derivatives have been the most widely used for the removal of F species [12,13,14,15,16,17,18,19,20,21,22]. However, some obvious issues such as poor stability and separation, high environmental susceptibility, and bad selectivity have limited the industrialization of these Al- and Mg-based materials [23,24,25,26].

In recent years, mixed-valent iron-based green rusts (GRs) have attracted attention for their efficient application in environmental remediation. GRs have the structural features of layered double hydroxides (LDHs) with the existence of amphoteric hydroxyl groups [27,28]. GRs can theoretically adsorb both inorganic anions and cationic metals. The adsorption process of different contaminants achieved by GRs is mainly attributed to several mechanisms, such as the redox process, surface complexation, and ion exchange [29,30]. Anionic contaminants are diffused and intercalated into Fe^II^–Fe^III^ interlayers during adsorption [31]. The adsorption properties and molecular structure of GRs make them a promising choice of adsorbent material for the removal of fluorinion [32,33,34,35]. Iron-based GRs can solve the issues of separation and environmental susceptibility to some extent but remain limited by poor selectivity and instability. Changes in pH can change the material characteristics of GRs (e.g., acid dissolution and alkaline flocculation), further negatively influencing their adsorption capacities [29,30].

The employment of cerium (Ce)-related oxides and hydroxides in attaining the high selectivity and adsorption enhancement of fluoride has been widely investigated. Xiaomei Wu et al. synthesized a Fe-Al-Ce tri-metal hydrous oxide to adsorb fluoride and found that Ce–OH was the preferential adsorption site within a specific F concentration range [36]. Ankita Dhillon et al. intensified the removal of fluoride by a newly formed Ce–Zn binary metal oxide, exhibiting an adsorption capacity of 194 mg·g^−1^ at a pH of 7.0 [37]. Wen Tao et al. removed fluoride from wastewater by using oxalic acid-modified Ce-AlOOH and demonstrated that the Ce^IV^ species played an essential role in defluorination [38]. Therefore, (Ce)-related oxides and hydroxides have demonstrated the potential to enhance the absorption of F from wastewater. In substitution, Ce^IV^ can directly react with Fe^II^ to form Ce^III^ and Fe^III^, which may hinder the formation of the GR molecular structure. Therefore, in this study, Ce^IV^ was chosen to substitute Fe^II^ in the structures of GRs to synthesize a Fe^III^–Ce^IV^-based layered hydroxide composite (DD-LHC), aiming to solve the above-mentioned issues and achieve the development of new materials and the efficient removal of F anions from wastewater at the same time. “DD” in DD-LHC refers to double elements (Fe and Ce) with double valences (trivalent and tetravalent). To fully explore the adsorption characteristics of the new adsorbents toward fluoride, both DD-LHC and a control adsorbent of GR were prepared and employed in both batch and column modes. The effect of pH, concentration, and competing anions on the adsorption of F by DD-LHC and GR were analyzed in batch experiments. The column process was implemented in a continuous form and optimized using predictor and desirability functions in a central composite design (CCD). The influence of factors in the CCD was analyzed using surface responses. The adsorption kinetics in batch mode and the breakthrough curves in column mode under different pH conditions were further explored to obtain a full adsorption pattern. The reaction mechanisms of F^−^ adsorption onto DD-LHC were comprehensively investigated using various characterization strategies including Fourier transform infrared spectroscopy (FTIR), thermogravimetric analysis (TGA), X-ray diffraction (XRD), and scanning electron microscopy (SEM, HITACHI, H-9500, Tokyo, Japan). Research on highly selective and high-capacity adsorbent materials can help shorten process flows and simplify equipment configurations to some extent. This multi-scale analysis clarifies the dominant role of Ce–F coordination bonds and the extent of other mechanisms’ involvement, offering new insights into layered hydroxide composites’ adsorption behavior. This study not only introduces an alternative F-selected scavenger but also reveals the surface mechanisms underlying the heterogeneous adsorption of F anions by a Ce-based layered hydroxide composite.

## 2. Materials and Methods

### 2.1. Materials and the Synthesis of DD-LHC

Chemical reagents used for the preparation of the DD-LHC adsorbent and the adsorption experiments in different conditions are specified in the Supporting Information (SI). The deionized (DI) water used for all the further experiments was generated using a water purification device. All the relevant gadgets, glass containers and bottles were rinsed several times with DI water and dried using a vacuum drying oven before any experimental usage. DD-LHC samples with different molar ratios of Fe^III^ to Ce^IV^ were synthesized at room temperature using the coprecipitation method [28,31]. Specifically, Fe_3_(SO_4_)_2_ and Ce(SO_4_)_2_ were weighted at certain molar ratios of Fe^III^ to Ce^IV^ and then dissolved in DI water. NaOH was weighted at certain molar ratios of OH^−^ to Fe^III^ and dissolved. The two different solutions were fully mixed and mechanically stirred over 2 h. Hereafter, the mixture was centrifuged at 4000 rpm. The procedures for the dissolution and stirring were carried out in a vacuum glovebox to eliminate carbonate incorporation from the atmosphere into the interlayer space of the synthesized LDH. The precipitation was dried at 105 °C, manually milled, sieved (mesh size: 75 µm), and sealed for the following experiments. The point of zero charge was determined using the pH drift method [39]. The synthesis parameters and corresponding labels for each DD-LHC are presented in Table S1 in the SI. The control material of sulfate-intercalated green rust (i.e., GR(SO_4_^−2^)) was prepared at a molar ratio of Fe^II^ to Fe^III^ of 3.

### 2.2. Experimental Design

The stock solutions containing F^−^ anions were prepared by dissolving KF into DI water. 5M H_2_SO_4_ and 5M NaOH were titrated to adjust the pH of the stock solutions. The adsorption experiments in both batch mode and column mode across a wide pH range (1–13) were designed, carried out, and analyzed.

#### 2.2.1. Batch-Mode Adsorption Experiments

For the batch-mode equilibrium adsorption, DD-LHC and GR(SO_4_^−2^) were added to the stock solutions of 20–80 mM (mmol·L^−1^) at a solid-to-liquid ratio of 1.0 g·L^−1^, respectively, and stirred for over 60 min at ambient temperature. Four kinetic models, including the pseudo-first-order, pseudo-second-order, intraparticle diffusion, and Elovich models, were employed to determine the reaction rates and to analyze the potential reaction pathways [35]. The equations for the models and the corresponding specifications on variables are presented in SI (Equations (S1)–(S4)). For the analysis of selectivity, some competing anions including Br^−^, CI^−^, and NO_3_^−^ at 50 mM were selectively added to stock solutions at different pH conditions, respectively.

#### 2.2.2. Column-Mode Adsorption Experiments

The schematic diagram of the column-mode device has been specified in previous research [27]. A core cylindrical container accompanying two peristaltic pumps and a concentration-monitoring system were assembled to conduct the continuous process of removal. The CCD with three factors in 20 runs (8 cubic points, 6 center points in the cube, and 6 axial points) was set to analyze the influence of factors using response surface methodology (RSM) and to optimize the column-mode adsorption using a quadratic model predictor and desirability function (DF) [40,41,42]. RSM is commonly employed to reduce the number of experiments, minimize systematic errors, and pinpoint the interactions between every two CCD factors by multiple linear regression [43,44,45]. The factors and levels used are detailed in Table S2 in SI. Three factors including the initial concentration of influent (mM), the initial pH, and the flow rates (mL·min^−1^) responding to the breakthrough volumes (*V_bre_*) were deployed in the CCD. The breakthrough model of Thomas (Equation (S5), SI) was adopted to quantitatively evaluate column performance and verify the main adsorption mechanisms dominating the continuous process.

### 2.3. Analysis and Calculations

The concentrations of F^−^, Br^−^, Cl^−^, and NO_3_^−^ anions in the aqueous environment before and after the experiments were all measured using an ion meter (PXSJ-216F, INESA, Shanghai, China) to directly evaluate the adsorption performance (adsorption capabilities and selectivity) of the adsorbents. The different ion electrodes detected and the requirements (e.g., the measuring range, pH condition, the reference electrode model, and the calibration solution) for the detection process are specified in Table S3 in SI. The adsorption capacities (mg·g^−1^) of DD-LHC toward F contaminants at the equilibrium and at time *t* (i.e., *q*_e_ and *q*_t_) were calculated using Equations (1) and (2), respectively, where *V* is the volume (L) of the solution, *m* represents the mass amount (g) of DD-LHC, and c_0_, c_e_, and *c*_t_ are the concentrations of F^−^ in the stock solutions at the initial, equilibrium, and time *t*, respectively. The *V*_bre_ (L) was determined as the the total effluent volume over the flow time at a concentration of F^−^ lower than 0.02 mM. The desirability functions were applied to guide the prediction for the optimization of factors in the CCD by the quadratic optimizer [46]. The equation for the quadratic predictor is shown in Equation (3). The desirability functions and each variable of the predictor have been correspondingly explained in previous research. FTIR (Thermo Electron Co.-380FTIR, Waltham, MA, USA) was conducted over a wavenumber range from 400 to 4000 cm^−1^ at a step of 4 cm^−1^. TGA (STA 449F3, NETZSCH, Selb, Germany) was performed at a rate of 10 °C·min^−1^ over 30–1400 °C under an N_2_ atmosphere (GB2 12-9 1). The mineral phases of samples were determined by XRD (7000, Shimadzu, Kyoto, Japan) using Cu Kα radiation with 2θ of 5°–90°.

(1) $$q_{e}=\frac{\left(c_{0}-c_{e}\right)\times V}{m},$$

(2) $$q_{t}=\frac{\left(c_{0}-c_{t}\right)\times V}{m},$$

(3) $$Y=a_{0}+\sum_{i=1}^{n}a_{i}X_{i}+\sum_{i=1}^{n}a_{ii}x_{i}^{2}+\sum_{i=1}^{n}\sum_{j=i+1}^{n}a_{ij}X_{i}X_{j},$$

## 3. Results

### 3.1. Synthesis and Characterization of GR and DD-LHC

**Figure 1:**
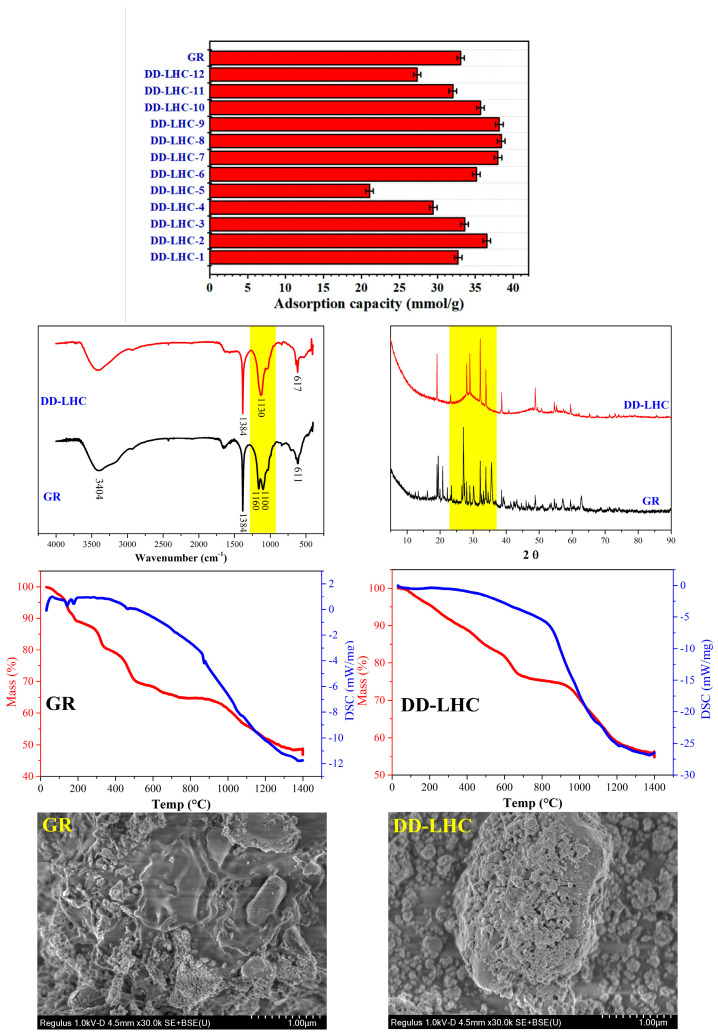
Adsorption capacities of GR (control group) and different DD-LHC samples affected by the synthesis parameters (experimental conditions: stock solution of 40 mM, initial pH of 7, DD-LHC-8) and characterization of GR and DD-LHC by FTIR, XRD, TGA, and SEM.

The adsorption capacities of GR (control group) and different DD-LHC samples affected by the synthesis parameters (experimental conditions: a stock solution of 40 mM, and initial pH of 7) and the characterization of GR and DD-LHC by FTIR, XRD, TGA, and SEM are shown in Figure 1. As seen, the adsorption capacities of the DD-LHC samples were strikingly influenced by the synthesis parameters. The maximum adsorption of 38.42 mmol·g^−1^ was obtained in the sample of DD-LHC-8, which was higher than that of the control group at 33.06 mmol·g^−1^. The substitution of Ce^IV^ for Fe^II^ in the GR structure potentially changed the reaction pathways of the layered double hydroxide to F anions [33,34,35]. The molar ratio of Fe^III^ to Ce^IV^ of 0.5 (DD-LHC-2), the concentration of Fe^III^ (M) of 0.75 (DD-LHC-7), and the molar ratio of OH^−^ to Fe^III^ of 8:1 (DD-LHC-8) were most favored in terms of facilitating the synthesis of DD-LHC samples with desirable adsorption capacities. Generally, a high dosage of both Fe^III^ and OH^−^ should be avoided, as they can deteriorate the adsorption process of DD-LHC toward F^−^ (Figure 1). The peaks at wavenumbers (cm^−1^) 3404, 1384, 1160, 1100, and 611 were formed in the FTIR spectrum of GR, which is consistent with previous research [39,47]. Comparatively, lesser adsorption peaks and a new band at 617 cm^−1^ were formed in the spectrum of DD-LHC. The new band is attributed to the formation of the Fe-O-Ce metal frame [36,48]. This band result can be attributed to the specific structural and compositional characteristics of our novel Fe^III^–Ce^IV^-based layered hydroxide composite. DD-LHC has a distinct molecular structure that combines layered hydroxides with a three-dimensional (3D) Fe-O-Ce metal frame. This unique structural environment can lead to variations in the vibrational modes of the Fe-O-Ce bonds, potentially causing the band to appear at a higher wavenumber than typically observed. In the XRD spectra, the peak characteristics of DD-LHC were different from those of GR, reflecting the influence of the Ce^IV^ to Fe^II^ substitution. A wide half-height width at 2θ of around 25–35 was observed in the DD-LHC sample, directly reflecting the existence of an amorphous state [30,31]. In the TGA curves, the pyrolysis process resulted in a mass loss of approximately 53% for GR and 45% for DD-LHC. Three small endothermic peaks of DSC at around 140, 179, and 461 °C were also detected for GR. DD-LHC demonstrated better structural stability than GR. The morphology of the layered hydroxides was observed in both samples. However, the classical intercalation of anions in GR was hardly found in DD-LHC. The two samples exhibited completely different surface morphologies. The three-dimensional (3D) structure of the metal frame was confirmed in DD-LHC. The XRD pattern for DD-LHC exhibits characteristic peaks, indicating the periodic stacking of positively charged metal hydroxide layers. The broadening of the peaks suggests a reduction in crystallite size, which is consistent with the amorphous nature observed in the SEM images. The absence of new mineral phases confirms that the adsorption of F^−^ is primarily a physical process involving coordination bonds and electrostatic attraction. The SEM images reveal distinct morphologies of GR and DD-LHC. While GR exhibits a classical intercalation of anions, DD-LHC shows a three-dimensional (3D) metal frame structure. This morphological difference is attributed to the substitution of Ce^IV^ for Fe^II^ in the GR structure, leading to the formation of the Fe-O-Ce framework. The 3D structure of DD-LHC is expected to enhance its adsorption performance by providing more active sites and facilitating the diffusion of F^−^ ions. The micromorphological results echoed the analysis of the other characterization strategies [49,50]. The molecular structure of DD-LHC combined the layered hydroxides with the 3D metal frame of Fe-O-Ce, which was different from the intercalated LDH of GR according to the characterization results. The difference in molecular structures between GR and DD-LHC verified the adsorption results.

### 3.2. Equilibrium Adsorption Experiment

#### 3.2.1. Effect of pH and Concentration on Adsorption

**Figure 2:**
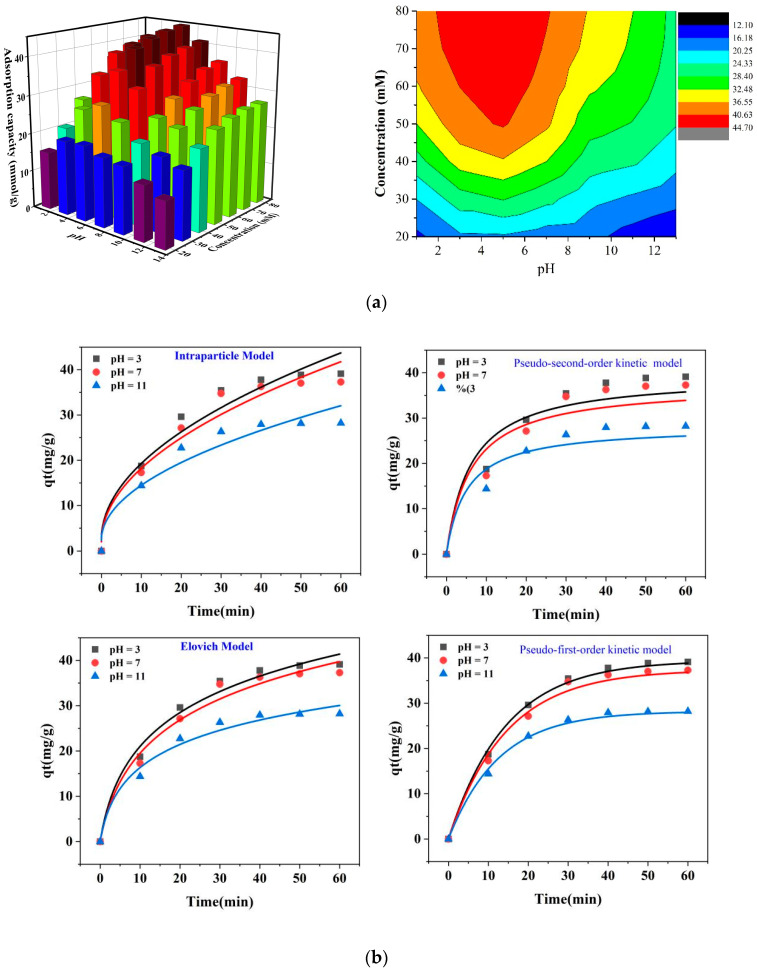

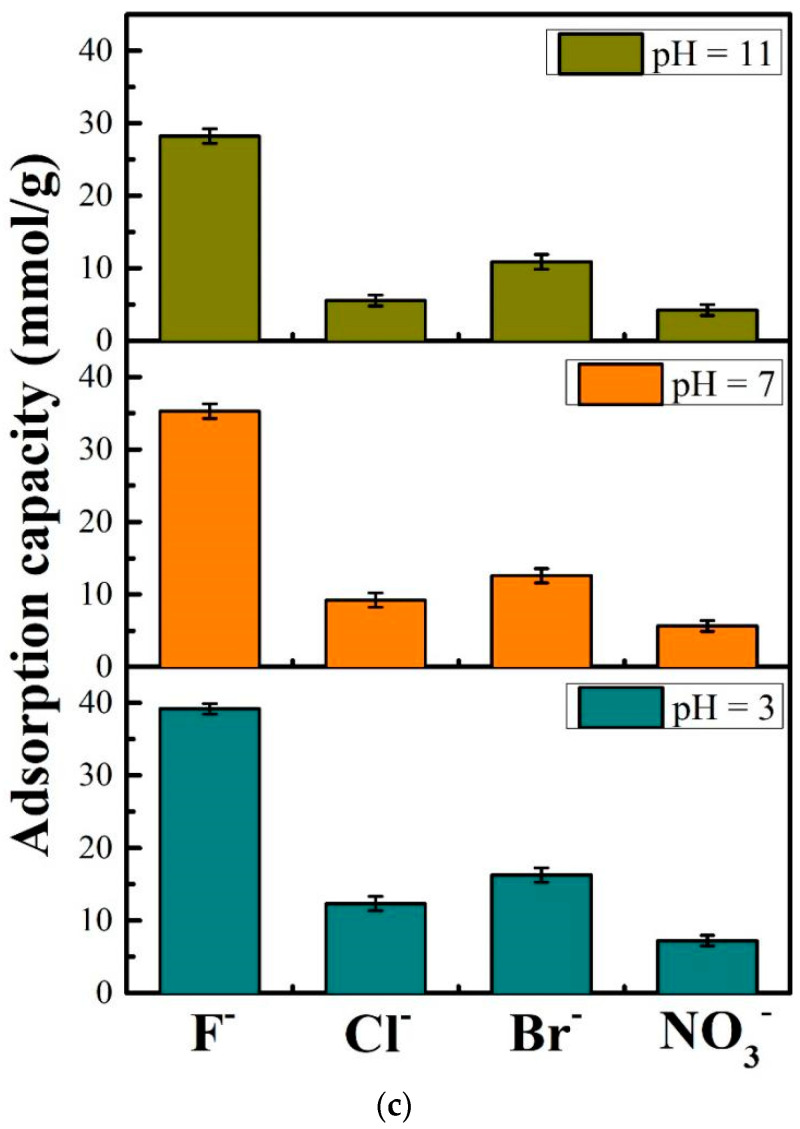
Equilibrium adsorption experiments and kinetics analysis, (**a**): effect of pH and concentrations on the adsorption of F^−^ by DD-LHC materials, (**b**): mathematical fittings of the kinetics models (experimental conditions: stock solution of 50 mM, initial pH of 3,7, and 11), (**c**): effect of some competing anions including Br^−^, CI^−^, and NO_3_^−^ on the adsorption of F^−^ by DD-LHC.

The effect of pH and concentration on the adsorption of F^−^ toward DD-LHC materials in the equilibrium adsorption experiments is shown in Figure 2a. The monovalent anion of F^−^ can maintain its form in a wide pH range due to the layout characteristics of its electrons [32,51]. The positively charged surface of DD-LHC is preferably adjusted, making it more conducive to the uptake of F^−^ to the DD-LHC [34,35,52]. Therefore, the environment with a pH below that corresponding to the point of zero charges (i.e., pH_zpc_) would facilitate the adsorption process, in theory [52,53]. As seen in Figure 2a, the adsorption of F^−^ by DD-LHC was explicitly affected by both pH and the initial concentration of F^−^ in the aqueous environment. The maximum adsorption capacity of DD-LHC to F^−^ of 44.68 mmol·g^−1^ was obtained at an initial pH of 5 and an initial concentration of 80 mM. The DD-LHC materials in acidic conditions achieved better adsorption compared to neutral and alkaline conditions. A gradually rising ridge and an inverted U-shaped peak at a pH around 5 (4–6) were observed in the 3D histogram and the contour diagram, with increasing initial concentration. A high initial concentration (50–80 mM) and a weakly acidic environment were most favored for the adsorption of F^−^ by DD-LHC. A high initial concentration was able to augment the effective utilization of active sites. A strong acidic environment was able to enhance the surface positivity of DD-LHC and was also able to exacerbate the partial dissolution loss of the adsorbent, which contrarily offset the positive effect of the surface charge and finally caused a decrease in the adsorption capacities of DD-LHC. The pH_pzc_ value for our material was found to be around pH 5. This value is consistent with the observed adsorption behavior, as the material exhibited better adsorption performance under acidic conditions (pH below pH_pzc_) where the surface was positively charged, facilitating the uptake of anions such as fluoride [43,44].

#### 3.2.2. Adsorption Kinetics Under Different pH Conditions

The mathematical fittings of the four kinetics models and the corresponding parameters of kinetics for the adsorption of F^−^ by DD-LHC materials are shown in Figure 2b and Table 1 [46], respectively. The accessibility of active sites determined by the matrices controls the adsorption kinetics in porous adsorbents [43,44]. As can be seen, the correlation coefficients (*Adj. R*^2^) of the pseudo-first-order kinetic model at the pHs of 3, 7, and 11 were 0.995, 0.982, and 0.976, respectively, all higher than the default α of 0.95. Moreover, the adsorption capacities calculated by the fitting model (i.e., *Cal. q_e_*, 39.76 mmol·g^−1^, 37.54 mmol·g^−1^, 28.30 mmol·g^−1^, Table 1) were close to the experimental results. Therefore, the pseudo-first-order kinetic model was suitable for the description of the adsorption process. The external/internal diffusion of F^−^ ions significantly influenced the heterogeneous adsorption of DD-LHC [54]. The electrostatic attraction influenced by the surface charge actively participated in the adsorption pathways. Comparatively, the three *Cal. q_e_* of the pseudo-second-order kinetic model were all greater than their experimental counterparts. The choice of a pseudo-second-order kinetic model for the fitting was therefore undesirable. The dominance of active sites for the adsorbent cannot adequately describe the adsorption process. The chemical valence of F anions was barely affected by the heterogeneous adsorption process. The differing fitting performance of the intraparticle diffusion and Elovich models across three different pH scenarios directly reflected the different reaction pathways. Generally, the adsorption conducted under acidic conditions (pH = 3) demonstrated better fitting results in the four kinetic models than in the other two circumstances (pH = 7 and 11). The acidic environment improves the surface positivity of DD-LHC, which facilitates the enhancement of heterogeneous adsorption. The excellent fitness of the Elovich model also verified the chemisorption of F^−^ by DD-LHC [55]. Therefore, in summary, several adsorption mechanisms such as electrostatic attraction, intraparticle diffusion, and surface complexation can be inferred to have imposed an influence on the adsorption of F^−^ by DD-LHC in an aqueous environment. The adjustment of pH altered the effect of different mechanisms during the whole reaction process.

#### 3.2.3. Selectivity Characteristics

The effect of some competing anions including Br^−^, CI^−^, and NO_3_^−^ on the adsorption of F^−^ toward DD-LHC is shown in Figure 2c. The adsorption capacities of DD-LHC of 39.18, 35.28, and 28.21 mmol·g^−1^ toward F^−^ ions were obtained at an initial pH of 3, 7, and 11, respectively. The existence of Br^−^, CI^−^, and NO_3_^−^ negatively influenced the uptake of F^−^ onto DD-LHC to some extent. Among the three anions, Br^−^ achieved the most significant effect on inhibiting the absorption of F^−^. The competitive order for the active sites of DD-LHC all followed Br^−^ > CI^−^ >NO_3_^−^ at a pH of 3, 7, and 11. The changes in pH barely affected the competitive order. Some intrinsic characteristics such as the ionic radius, the layout of the external electrons, hydrogen bonds, and redox potentially played a more vital role in determining the adsorption tendency. Despite the disturbing impact of the competing anions, the adsorption capacities of F^−^ of DD-LHC still far exceeded the competing anions. The selectivity of DD-LHC for F^−^ was specific and distinct across a wide pH range.

### 3.3. Optimization for F Removal in Column Mode

**Figure 3:**
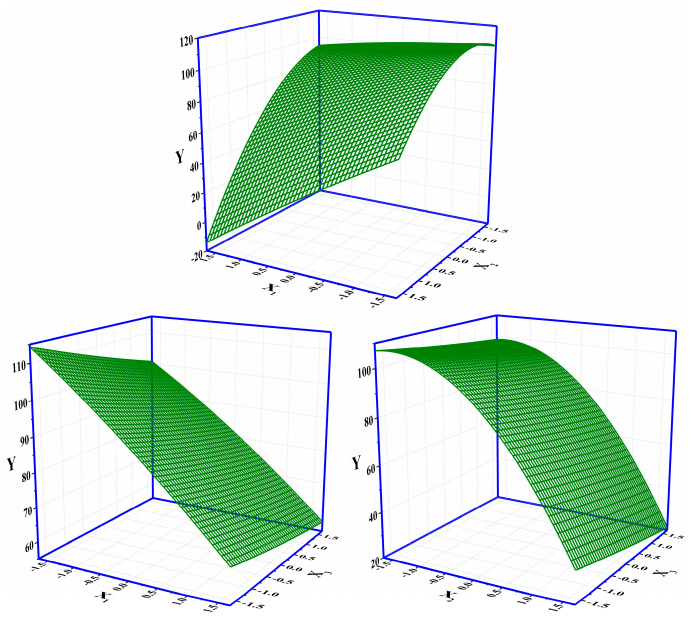
Surface responses influenced by X_1_–X_2_, X_1_–X_3_, and X_2_–X_3_ in the CCD (X_1_: the initial concentration of influent (mM); X_2_: the initial pH; X_3_: the flow rate (mL·min^−1^)).

**Figure 4:**
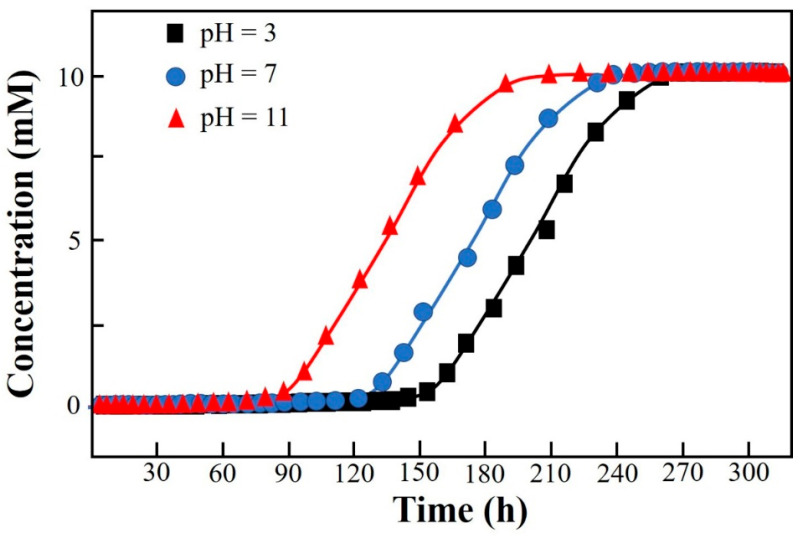
Breakthrough curves of the Thomas model under different pH conditions in column mode.

The layout of the CCD and the corresponding results of the response are shown in Table 2. The significance analysis in the CCD layout (response: *V_bre_*(L)) is shown in Table S4 in SI. The maximum response of 112.34 L among 20 runs was obtained under the condition of X_1_(−1)X_2_(−1)X_3_(−1) (i.e., an initial concentration of influent of 10 mM, an initial pH of 4, and a flow rate of 10 mL·min^−1^) (Table 2). The *Adj. R*^2^ of the CCD was 99.08%, indicating that the whole significance analysis was reliable (Table S4, SI). The significance probabilities (*p* values) of the regressions (i.e., linear, square, and interaction) were all 0, lower than the default 0.05. The changes in the three parameters in the CCD all had a significant effect on the column-mode adsorption of F^−^. Specifically, the *Adj. R*^2^ values of X_1_*X_2_, X_1_*X_3_, and X_2_*X_3_ were 0.000, 0.518, and 0.623, respectively. The interaction effect between X_1_ and X_2_ was significant. The adjustment of X_1_ would influence the setting of X_2_ and vice versa. The Pareto chart showing the standardized effects of the regressions in the CCD is shown in Figure S1 in SI. The descending ranking of significance was as follows: X_2_ > X_1_ > X_2_X_2_ > X_1_X_2_ > X_3_ > X_1_X_1_ > X_1_X_3_ > X_2_X_3_ > X_3_X_3_. The regressions including X_2_, X_1_, X_2_X_2_, X_1_X_2_, and X_3_ were remarkable. It is clear that the adjustment of the initial pH (X_2_) should be extra cautious and valued as it not only had a direct influence on the removal of F^−^ by DD-LHC in column mode but also indirectly interfered with the role of the initial concentration (X_2_). Surface responses influenced by X_1_–X_2_, X_1_–X_3_, and X_2_–X_3_ in the CCD are shown in Figure 3. Surface responses affected by the three groups were observable and different in general. The interaction between X_1_ and X_2_ was verified. The changes in the aqueous conditions (pH and concentration) hardly affected the adsorption of DD-LHC toward F^−^ anions controlled by the flow rate (X_1_–X_3_ and X_2_–X_3_). Therefore, the flow rate of the wastewater was independently adjustable regardless of the aqueous conditions. The optimization of the CCD by the quadratic predictor (Equation (4)) and the desirability function is shown in Figure S2 in SI. The desirability (d) value was 1, meaning that the fitting process was reliable and acceptable. The maximum response of 118.90 L was obtained by the quadratic predictor. X_1_(−1.682)X_2_(−0.765)X_3_(−1.682) was determined as the optimal combination of factors, specifically, including an initial concentration of influent (mM) of 6.59, an initial pH of 4.71, and a flow rate (mL·min^−1^) of 8.30. The breakthrough curves of the Thomas model under different pH conditions in column mode were fitted after the optimization process. As seen in Figure 4, the fittings of the Thomas model at pH 3, 7, and 11 were significant (R^2^ = 0.992, 0.996, and 0.998). The Thomas model can be used to an extent to predict the breakthrough curve for the adsorption and ion exchange of F^−^ ions from the continuous liquids by the DD-LHC in column mode. The kth values for the curves obtained at an initial pH of 3, 7, and 11 were 0.0083, 0.0121, and 0.0148 mL·mmol^−1^·min^−1^, respectively, meaning the acidic environment led to a more pronounced adsorption performance than that procured in neutral and alkaline environments. The adsorption process of DD-LHC toward F^−^ anions was dominated by one main nonchemical pathway and several potential auxiliary mechanisms.

The regression equation in uncoded units can be written as follows:

Y = 86.166 − 13.599 *X*_1_ − 22.320 *X*_2_ − 3.156 *X*_3_ − 0.736 *X*_1_ × *X*_1_ − 7.568 *X*_2_ × *X*_2_ + 0.185 *X*_3_ × *X*_3_ − 5.853 *X*_1_ × *X*_2_ + 0.537 *X*_1_ × *X*_3_ − 0.407 *X*_2_ × *X*_3_, (4)

## 4. Discussion

### 4.1. Mechanism Explorations

**Figure 5:**
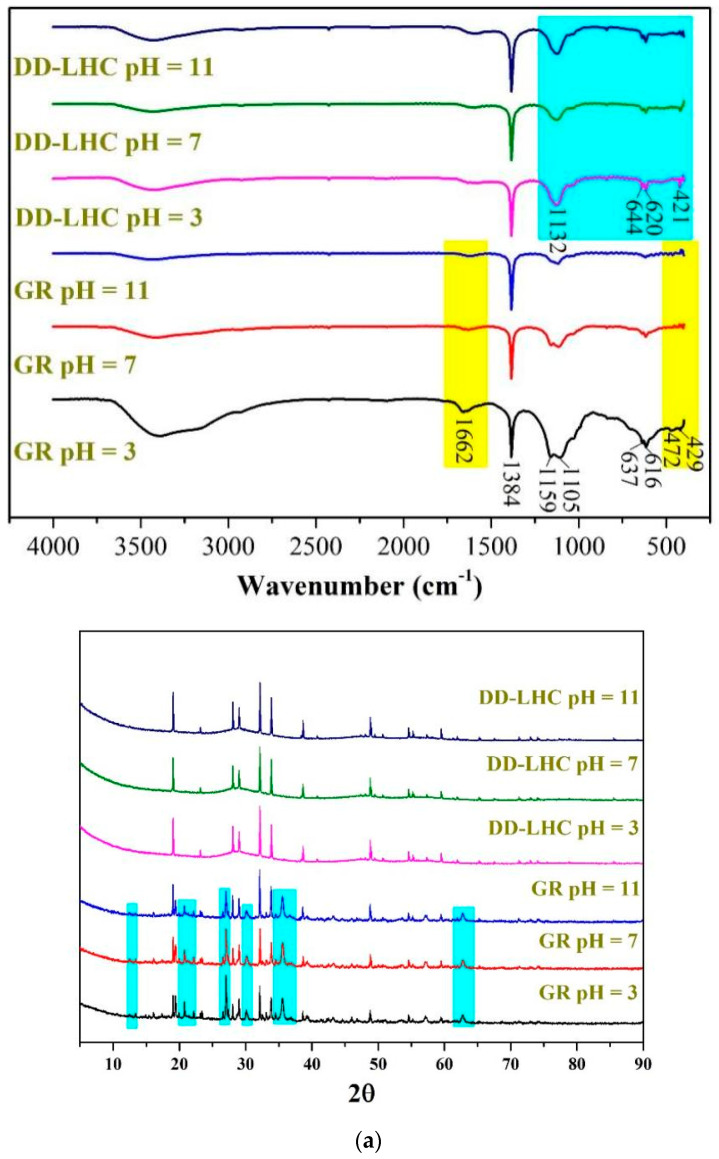

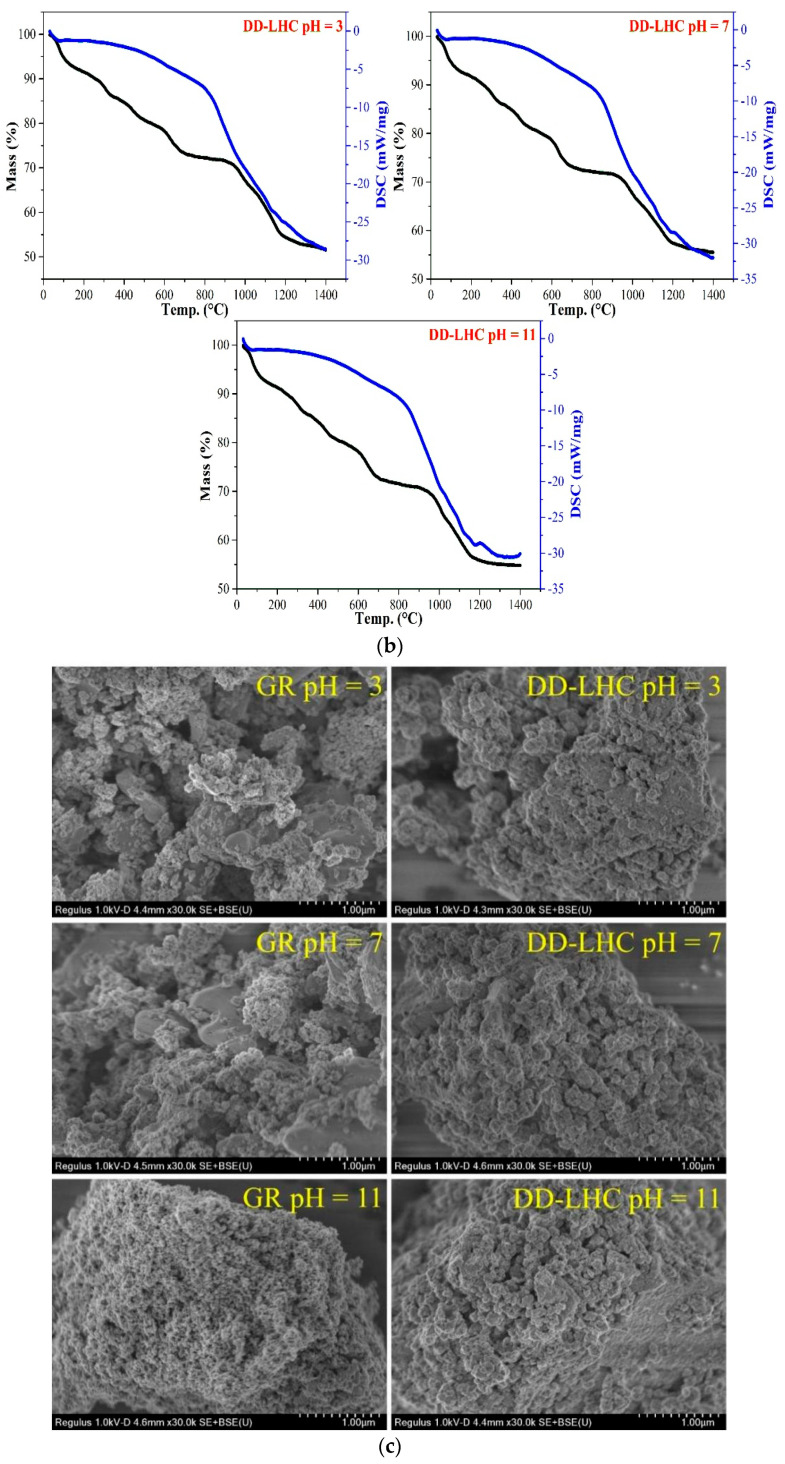
Comprehensive characterization of GR and DD-LHC samples under different pH conditions following equilibrium adsorption experiments, (**a**): FITR and XRD spectra of GR and DD-LHC samples; Blue: DD-LHC-specific peaks; Yellow: GR bands at pH 3 (**b**): TGA curves of GR and DD-LHC samples; (**c**): TGA curves of GR and DD-LHC samples.

The FTIR and XRD spectra, the TGA curves, and the SEM images of GR and DD-LHC samples under different pH conditions (3, 7, and 11) following the equilibrium adsorption experiments are shown in Figure 5. As seen in Figure 5a, some bands at approximately 1662, 1384, 1159, 1105, 637, 616, 472, and 429 cm^−1^ were formed in the spectrum of GR at a pH of 3 following the experiments. Several peaks including 637, 472, and 429 cm^−1^ disappeared when the pH reached 7 and 11. The characteristics of the spectra of GR were similar to analyses in previous research [27,31], indicating potentially similar adsorption pathways (i.e., electrostatic attraction and ion exchange). The change in pH significantly influenced the reaction mechanisms of GR toward F^−^. More peaks at 644, 620, and 421 cm^−1^ were observed in the spectrum of DD-LHC compared to the spectrum of the original sample (Figure 1), which directly reflects the adsorption of F^−^. The spectra of DD-LHC at pH 3, 7, and 11 were similar. The change in pH had a minor effect on the stability and adsorption pathways of DD-LHC. Neither the XRD spectra of the GR nor the DD-LHC samples changed after the experiments, which indicated that there were no new mineral phases formed during the adsorption process. The adsorption of F^−^ by GR and DD-LHC was achieved mainly by physical reactions and coordination bonds. As seen in Figure 5b, approximately 53%, 57%, and 58% of GR and 49%, 45%, and 45% of DD-LHC samples at the pHs of 3, 7, and 11 were lost during thermal decomposition. Comparatively, lesser mass losses were demonstrated in DD-LHC, which not only echoes the higher adsorption results achieved in DD-LHC (Figure 1) but also verifies the different main reactions for the removal of F^−^ anions at different pH conditions. The TGA results were in agreement with the FTIR analysis in general. As seen in Figure 5c, different adsorption morphologies were observed in GR and DD-LHC. It is clear that DD-LHC demonstrated better material stability under different pH conditions than GR, especially at a pH of 3 and 7. DD-LHC avoided the adsorption loss caused by the dissolution and flocculation of Fe-based materials in acid and alkaline environments to some extent. Similar results of morphologies for a range of adsorbents (e.g., ligand-based sustainable composite material, tea waste-based natural adsorbent, functionalized layered double hydroxides composite bio-adsorbent, agrowaste-based functional green adsorbents, and Mg/Al-LDH composite materials) and heavy metal ions (e.g., nickel(II), copper(II), chromium(VI), Cd(II), and Pb(II)) echoing the current investigation have been analyzed in some studies [56,57,58,59,60]. The nanoscale particles formed on the adsorbents of GR and DD-LHC had different morphologies, visually indicating the different potential main reaction pathways.

### 4.2. Comprehensive Discussion on Removal Mechanisms

For the application of different GR materials, mechanisms including electrostatic attraction, ion exchange, intraparticle transition, and reduction have been concluded to be the four main pathways for the removal of different contaminants by many studies [28,61,62]. The substitution of Ce^IV^ for Fe^II^ in the intercalated layered structure of GR potentially changed the reaction pathways for F^−^ removal, which are typically dominated by LDH materials composed of Fe^II^–Fe^III^ (Figure 1). The 3D metal frame of Fe-O-Ce was confirmed as the molecular structure of DD-LHC based on the characterization results (Figure 1), which demonstrated better adsorption results and material stability in comparison with GR (Figure 1 and Figure 5b,c). The adsorption mechanisms including electrostatic attraction, intraparticle diffusion, and chemical complexation were mathematically inferred by the fittings of the four kinetics models (Table 1 and Figure 2) [63,64,65,66]. However, new mineral phases could not be determined from the XRD spectra of the DD-LHC samples (Figure 5b). The physical reactions and coordination bonds were demonstrated to be the more reliable pathways for the adsorption of F^−^ by DD-LHC (Figure 5a,b). The results of the breakthrough curves quantitatively asserted the invalidation of the potential chemical reaction participating in the process of F^−^ removal (Figure 4). The coordination bonds for the complexation of Ce–F have been reported in previous research [10,36,37]. Furthermore, the different surface morphologies of the GR and DD-LHC samples ruled out the dominant influence caused by GR-related physical mechanisms and demonstrated the primary role of the Ce–F coordination bonds (Figure 5c). Generally, the adsorption process of DD-LHC toward F^−^ anions was controlled by the Ce–F coordination bonds and influenced by potential auxiliary mechanisms including electrostatic attraction, ion exchange (substitution), and intraparticle transition (Figure 1, Figure 3, Figure 4 and Figure 5 and Table 1).

## 5. Conclusions

The molar ratio of Fe^III^ to Ce^IV^ of 0.5, the concentration of Fe^III^ (M) of 0.75, and the molar ratio of OH^−^ to Fe^III^ of 8:1 were the favored parameters in facilitating the synthesis of DD-LHC samples. A high dosage of both Fe^III^ and OH^−^, which deteriorates the adsorption process, should be avoided. DD-LHC exhibited a molecular structure of layered hydroxides combined with a 3D metal frame of Fe-O-Ce. A high initial concentration and a weakly acidic environment were most beneficial for the adsorption of F^−^ by DD-LHC. An excessively acidic environment can enhance the surface positivity of DD-LHC while also exacerbating the partial dissolution loss of the adsorbent. The pseudo-first-order kinetic model suitably described the whole adsorption process. The adsorption carried out under acidic conditions achieved better fitting results with the kinetics models than that carried out in neutral and alkaline conditions. The selectivity of DD-LHC for F^−^ was evident across a wide pH range. The adjustment of the initial pH not only had a direct influence on the removal of F^−^ by DD-LHC but also indirectly interfered with the role of the initial concentration in column mode. X_1_(−1.682)X_2_(−0.765)X_3_(−1.682) were confirmed as the optimal combination of factors for the column-mode continuous process. The adsorption process of DD-LHC toward F^−^ anions was controlled by Ce–F coordination bonds and influenced by electrostatic attraction, ion substitution, and intraparticle transition to varying extents.

## Figures and Tables

**Table 1:** Parameters of kinetics for the adsorption of F^−^ by DD-LHC materials.

Models	Parameters	pH		
		3	7	11
Pseudo-first-order kinetic model $\ln\left(q_{e}-q_{t}\right)=\ln q_{e}-k_{1}t$	*k*_1_ (×10^−2^, min^−1^)	6.999	6.965	7.97
	*Cal. q_e_* (mmol·g^−1^)	39.44	37.39	28.21
	*Adj. R* ^2^	0.998	0.992	0.996
Pseudo-second-order kinetic model $\frac{t}{q_{t}}=\frac{1}{k_{2}q_{e}^{2}}+\frac{t}{q_{e}}$	*k*_2_ (×10^−3^, g·mg^−1^·min^−1^)	4.13	4.33	6.94
	*Cal. q_e_* (mmol·g^−1^)	39.44	37.39	28.25
	*Adj. R* ^2^	0.938	0.923	0.936
Intraparticle model $q_{t}=k_{p}t^{0.5}+C$	*k_p_* (mmol·g^−1^·min^−0.5^)	5.334	5.139	3.837
	*C*	2.408	1.961	2.332
	*Adj. R* ^2^	0.955	0.952	0.935
Elovich model $q_{t}=\frac{1}{\beta }\ln\left(\alpha \beta \right)+\frac{1}{\beta }\ln\left(t\right)$	*α* (mg·g^−1^ min^−1^)	5.427	4.63	5.184
	*β* (×10^−2^, g·mg^−1^)	7.954	7.878	12.178
	*Adj. R* ^2^	0.985	0.98	0.98

**Table 2:** Layout of CCD and the corresponding results of the response.

No.	Initial Concentration of Influent (mM)	Initial pH	Flow Rates (mL·min^−1^)	Response
	*X*_1_	*X*_2_	*X*_3_	*V_bre_* (L)
1	−1 (10)	−1 (4)	−1 (10)	112.34
2	1 (20)	−1	−1	97.67
3	−1	1 (10)	−1	78.51
4	1	1	−1	40.28
5	−1	−1	1 (15)	105.06
6	1	−1	1	92.39
7	−1	1	1	69.45
8	1	1	1	33.52
9	−1.682 (6.59)	0 (7)	0 (12.5)	108.27
10	1.682 (23.41)	0	0	58.19
11	0 (15)	−1.682 (1.95)	0	99.32
12	0	1.682 (23.41)	0	28.49
13	0	0	−1.682 (8.30)	90.21
14	0	0	1.682 (16.71)	81.46
15	0	0	0	85.32
16	0	0	0	87.49
17	0	0	0	84.93
18	0	0	0	85.17
19	0	0	0	88.36
20	0	0	0	86.02

## Data Availability

The original contributions presented in this study are included in the article and Supplementary Materials. Further inquiries can be directed to the corresponding authors.

## Supplementary Materials

The following supporting information can be downloaded at: https://www.mdpi.com/article/10.3390/ma18112665/s1, Figure S1: Pareto chart of the standardized effects; Figure S2: Optimization of the CCD by the quadratic predictor and the desirability function; Table S1: Parameters and labels for the synthesis of DD-LHC; Table S2: Factors and levels in the CCD (α = 1.682); Table S3: Choices of ion electrodes and the requirements for the detection of F^−^, Br^−^, Cl^−^, and NO_3_^−^; Table S4: Significance analysis in the CCD layout (Response: *V_bre_* (L)).

## References

[1] Gan C.D., Jia Y.B., Yang J.Y. (2021). Remediation of fluoride contaminated soil with nano-hydroxyapatite amendment: Response of soil fluoride bioavailability and microbial communities. J. Hazard. Mater.

[2] Singh G., Kumari B., Sinam G., Kriti, Kumar N., Mallick S. (2018). Fluoride distribution and contamination in the water, soil and plants continuum and its remedial technologies, an Indian perspective – a review. Environ. Pollut..

[3] Shu J.C., Chen M.J., Wu H.P., Li B.B., Wang B., Li B., Liu R.L., Liu Z.H. (2019). An innovative method for synergistic stabilization/solidification of Mn^2+^, NH^4+^-N, PO_4_^3−^ and F^−^ in electrolytic manganese residue and phosphogypsum. J. Hazard. Mater..

[4] Castaneda L.F., Coreno O., Nava J.L., Carreno G. (2020). Removal of fluoride and hydrated silica from underground water by electrocoagulation in a flow channel reactor. Chemosphere.

[5] Yue B.J., Zhang X.H., Li W.P., Wang J.D., Sun Z.L., Niu R.Y. (2020). Fluoride exposure altered metabolomic profile in rat serum. Chemosphere.

[6] Rashid U.S., Bezbaruah A.N. (2020). Citric acid modified granular activated carbon for enhanced defluoridation. Chemosphere.

[7] Abtahi M., Koolivand A., Dobaradaran S., Yaghmaeian K., Mohseni-Bandpei A., Khaloo S.S., Jorfi S., Saeedi R. (2018). Defluoridation of synthetic and natural waters by polyaluminum chloride-chitosan (PACl-Ch) composite coagulant. Water Sci. Technol.-Water Supply.

[8] Pang T., Chan T.S.A., Jande Y.A.C., Shen J.J. (2020). Removal of fluoride from water using activated carbon fibres modified with zirconium by a drop-coating method. Chemosphere.

[9] Min X.B., Zhu M.F., He Y.J., Wang Y.Y., Deng H.Y., Wang S., Jin L.F., Wang H.Y., Zhang L.Y., Chai L.Y. (2020). Selective removal of Cl^−^ and F^−^ from complex solution via electrochemistry deionization with bismuth/reduced graphene oxide composite electrode. Chemosphere.

[10] He J.J., Xu Y.H., Xiong Z.K., Lai B., Sun Y., Yang Y., Yang L.W. (2020). The enhanced removal of phosphate by structural defects and competitive fluoride adsorption on cerium-based adsorbent. Chemosphere.

[11] Hegde R.M., Rego R.M., Potla K.M., Kurkuri M.D., Kigga M. (2020). Bio-inspired materials for defluoridation of water: A review. Chemosphere.

[12] Luo C.H., Tian J., Zhu P.L., Zhou B., Bu D., Lu X.B. (2018). Simultaneous removal of fluoride and arsenic in geothermal water in Tibet using modified yak dung biochar as an adsorbent. Roy. Soc. Open Sci..

[13] Etawi H., Al-Rawajfeh A.E., Al-Ma A., Al-Amaireh M.N., Alfwaeer R.N., Al-Hawamdeh S., Al Dwairi R.A., Ababneh S.A. (2018). Efficiency and mechanism of water defluoridation by mixtures of Jordanian Zeolite, Pozzolana, Feldspar, and Tripoli. Desalin Water Treat..

[14] Bibi S., Farooqi A., Yasmin A., Kamran M.A., Niazi N.K. (2017). Arsenic and fluoride removal by potato peel and rice husk (PPRH) ash in aqueous environments. Int. J. Phytoremediation.

[15] Cho D.W., Han Y.S., Lee J., Jang J.Y., Yim G.J., Cho S., Lee J.S., Cheong Y.W. (2020). Water defluorination using granular composite synthesized via hydrothermal treatment of polyaluminum chloride (PAC) sludge. Chemosphere.

[16] Mejia G.V., Solache-Rios M., Martinez-Miranda V. (2017). Removal of fluoride and arsenate ions from aqueous solutions and natural water by modified natural materials. Desalin. Water Treat..

[17] Jadhav S.V., Bringas E., Yadav G.D., Rathod V.K., Ortiz I., Marathe K.V. (2015). Arsenic and fluoride contaminated groundwaters: A review of current technologies for contaminants removal. Environ. Manag..

[18] Teutli-Sequeira A., Solache-Rios M., Martinez-Miranda V., Linares-Hernandez I. (2015). Behavior of fluoride removal by aluminum modified zeolitic tuff and hematite in column systems and the thermodynamic parameters of the process. Water Air Soil. Poll..

[19] Prathna T.C., Sitompul D.N., Sharma S.K., Kennedy M. (2018). Synthesis, characterization and performance of iron oxide/alumina-based nanoadsorbents for simultaneous arsenic and fluoride removal. Desalin. Water Treat..

[20] Mejia G.V., Martinez-Miranda V., Fall C., Linares-Hernandez I., Solache-Rios M. (2015). Comparison of Fe–Al-modified natural materials by an electrochemical method and chemical precipitation for the adsorption of F^−^ and As(V). Environ. Technol..

[21] Wu L.P., Lin X.Y., Zhou X.B., Luo X.G. (2016). Removal of uranium and fluorine from wastewater by double-functional microsphere adsorbent of SA/CMC loaded with calcium and aluminum. Appl. Surf. Sci..

[22] Oladoja N.A., Hu S., Drewes J.E., Helmreich B. (2016). Insight into the defluoridation efficiency of nano magnesium oxide in groundwater system contaminated with hexavalent chromium and fluoride. Sep. Purif. Technol..

[23] Zhou G., Meng Q., Li S., Song R., Wang Q., Xu Z., Xing Z. (2022). Novel magnetic metal-organic framework derivative: An adsorbent for efficient removal of fluorine-containing wastewater in mines. J. Environ. Chem. Eng..

[24] Yang Y., Li X., Gu Y., Lin H., Jie B., Zhang Q., Zhang X. (2022). Adsorption property of fluoride in water by metal organic framework: Optimization of the process by response surface methodology technique. Surf. Interfaces.

[25] Lv J.-F., Zheng Y.-X., Tong X., Li X. (2021). Clean utilization of waste rocks as a novel adsorbent to treat the beneficiation wastewater containing arsenic and fluorine. J. Clean. Prod..

[26] Wang Z., Gu X., Zhang Y., Zhang X., Ngo H.H., Liu Y., Jiang W., Tan X., Wang X., Zhang J. (2021). Activated nano-Al_2_O_3_ loaded on polyurethane foam as a potential carrier for fluorine removal. J. Water Process Eng..

[27] Huang T., Zhang S.W., Zhou L.L., Liu L.F. (2021). Electrokinetics couples with the adsorption of activated carbon-supported hydroxycarbonate green rust that enhances the removal of Sr cations from the stock solution in batch and column. Sep. Purif. Technol..

[28] Huang T., Zhang S.-W., Xie J., Zhou L., Liu L.-F. (2021). Effective adsorption of quadrivalent cerium by synthesized laurylsulfonate green rust in a central composite design. J. Environ. Sci..

[29] Bhave C., Shejwalkar S. (2018). A review on the synthesis and applications of green rust for environmental pollutant remediation. Int. J. Environ. Sci. Te.

[30] Usman M., Byrne J.M., Chaudhary A., Orsetti S., Hanna K., Ruby C., Kappler A., Haderlein S.B. (2018). Magnetite and Green Rust: Synthesis, Properties, and Environmental Applications of Mixed-Valent Iron Minerals. Chem. Rev..

[31] Huang T., Su Z., Dai Y., Zhou L. (2021). Enhancement of the heterogeneous adsorption and incorporation of uraniumVIcaused by the intercalation of β-cyclodextrin into the green rust. Environ. Pollut..

[32] Collivignarelli M.C., Bellazzi S., Caccamo F.M., Calatroni S., Milanese C., Baldi M., Abbà A., Sorlini S., Bertanza G. (2023). Removal of Per- and Polyfluoroalkyl Substances by Adsorption on Innovative Adsorbent Materials. Sustainability.

[33] Kameda T., Oba J., Yoshioka T. (2015). Recyclable Mg–Al layered double hydroxides for fluoride removal: Kinetic and equilibrium studies. J. Hazard. Mater..

[34] Kang D., Yu X., Tong S., Ge M., Zuo J., Cao C., Song W. (2013). Performance and mechanism of Mg/Fe layered double hydroxides for fluoride and arsenate removal from aqueous solution. Chem. Eng. J..

[35] Najdanović S.M., Petrović M.M., Kostić M.M., Mitrović J.Z., Bojić D.V., Antonijević M.D., Bojić A.L. (2020). Electrochemical synthesis and characterization of basic bismuth nitrate [Bi_6_O_5_(OH)_3_](NO_3_)_5_·2H_2_O: A potential highly efficient sorbent for textile reactive dye removal. Res. Chem. Intermed..

[36] Wu X., Zhang Y., Dou X., Zhao B., Yang M. (2013). Fluoride adsorption on an Fe–Al–Ce trimetal hydrous oxide: Characterization of adsorption sites and adsorbed fluorine complex species. Chem. Eng. J..

[37] Dhillon A., Soni S.K., Kumar D. (2017). Enhanced fluoride removal performance by Ce–Zn binary metal oxide: Adsorption characteristics and mechanism. J. Fluorine Chem..

[38] Tao W., Zhong H., Pan X., Wang P., Wang H., Huang L. (2020). Removal of fluoride from wastewater solution using Ce-AlOOH with oxalic acid as modification. J. Hazard. Mater..

[39] Huang T., Zhang S.W., Liu L.F., Zhou L.L. (2021). Green rust functionalized geopolymer of composite cementitious materials and its application on treating chromate in a holistic system. Chemosphere.

[40] Gholami Z., Azqhandi M.H.A., Sabzevari M.H., Khazali F. (2023). Evaluation of least square support vector machine, generalized regression neural network and response surface methodology in modeling the removal of Levofloxacin and Ciprofloxacin from aqueous solutions using ionic liquid @Graphene oxide@ ionic liquid NC. Alex. Eng. J..

[41] Deylami S., Sabzevari M.H., Ghaedi M., Azqhandi M.H.A., Marahel F. (2023). Efficient photodegradation of disulfine blue dye and Tetracycline over Robust and Green g-CN/Ag_3_VO_4_/PAN nanofibers: Experimental design, RSM, RBF-NN and ANFIS modeling. Process Saf. Environ..

[42] Omidi M.H., Azqhandi M.H.A., Ghalami-Choobar B. (2022). Synthesis, characterization, and application of graphene oxide/layered double hydroxide/poly acrylic acid nanocomposite (LDH-rGO-PAA NC) for tetracycline removal: A comprehensive chemometric study. Chemosphere.

[43] Velinov N., Radović Vučić M., Petrović M., Najdanović S., Kostić M., Mitrović J., Bojić A. (2023). The influence of various solvents’ polarity in the synthesis of wood biowaste sorbent: Evaluation of dye sorption. Biomass Convers. Biorefinery.

[44] Naderi K., Foroughi M., Azqhandi M.H.A. (2022). Tetracycline capture from aqueous solutions by nanocomposite of MWCNTs reinforced with glutaraldehyde cross-linked poly (vinyl alcohol)/chitosan. Chemosphere.

[45] Ghaedi M., Mazaheri H., Khodadoust S., Hajati S., Purkait M.K. (2015). Application of central composite design for simultaneous removal of methylene blue and Pb^2+^ ions by walnut wood activated carbon. Spectrochim. Acta Part A Mol. Biomol. Spectrosc..

[46] Najdanović S.M., Kostić M.M., Petrović M.M., Velinov N.D., Radović Vučić M.D., Mitrović J.Z., Bojić A.L. (2025). Effect of Electrochemical Synthesis Parameters on the Morphology, Crystal and Chemical Structure, and Sorption Efficiency of Basic Bismuth Nitrates. Molecules.

[47] Huang T., Song D., Zhou L., Tao H., Li A., Zhang S.-W., Liu L.-F. (2022). Non-thermal plasma irradiated polyaluminum chloride for the heterogeneous adsorption enhancement of Cs^+^ and Sr^2+^ in a binary system. J. Hazard. Mater..

[48] Zhang T., Li Q., Xiao H., Mei Z., Lu H., Zhou Y. (2013). Enhanced fluoride removal from water by non-thermal plasma modified CeO2/Mg–Fe layered double hydroxides. Appl. Clay Sci..

[49] Kubra K.T., Hasan M.M., Hasan M.N., Salman M.S., Khaleque M.A., Sheikh M.C., Rehan A.I., Rasee A.I., Waliullah R.M., Awual M.E. (2023). The heavy lanthanide of Thulium(III) separation and recovery using specific ligand-based facial composite adsorbent. Colloids Surf. A Physicochem. Eng. Asp..

[50] Velinov N., Najdanovic S., Vucic M.R., Mitrovic J., Kostic M., Bojic D., Bojic A. (2019). Biosorption of Loperamide by Cellulosic-Al2O3 Hybrid: Optimization, Kinetic, Isothermal and Thermodynamic Studies. Cellul. Chem. Technol..

[51] Haldar D., Duarah P., Purkait M.K. (2020). MOFs for the treatment of arsenic, fluoride and iron contaminated drinking water: A review. Chemosphere.

[52] Lv L., He J., Wei M., Evans D.G., Zhou Z. (2007). Treatment of high fluoride concentration water by MgAl-CO_3_ layered double hydroxides: Kinetic and equilibrium studies. Water Res..

[53] Rochette C.N., Crassous J.J., Drechsler M., Gaboriaud F., Eloy M., de Gaudemaris B., Duval J.F.L. (2013). Shell Structure of Natural Rubber Particles: Evidence of Chemical Stratification by Electrokinetics and Cryo-TEM. Langmuir.

[54] Khomeyrani S.F.N., Ghalami-Choobar B., Azqhandi M.H.A., Foroughi M. (2022). An enhanced removal of para-nitrophenol (PNP) from water media using CaAl-layered double hydroxide-loaded magnetic g-CN nanocomposite. J. Water Process Eng..

[55] Wang J., Guo X. (2020). Adsorption kinetic models: Physical meanings, applications, and solving methods. J. Hazard. Mater..

[56] Awual M.R., Hasan M.M., Iqbal J., Islam M.A., Islam A., Khandaker S., Asiri A.M., Rahman M.M. (2020). Ligand based sustainable composite material for sensitive nickel(II) capturing in aqueous media. J. Environ. Chem. Eng..

[57] Kabir M.M., Mouna S.S.P., Akter S., Khandaker S., Didar-ul-Alam M., Bahadur N.M., Mohinuzzaman M., Islam M.A., Shenashen M.A. (2021). Tea waste based natural adsorbent for toxic pollutant removal from waste samples. J. Mol. Liq..

[58] Khandaker S., Hossain M.T., Saha P.K., Rayhan U., Islam A., Choudhury T.R., Awual M.R. (2021). Functionalized layered double hydroxides composite bio-adsorbent for efficient copper (II) ion encapsulation from wastewater. J. Environ. Manag..

[59] Kabir M.M., Akter M.M., Khandaker S., Gilroyed B.H., Didar-ul-Alam M., Hakim M., Awual M.R. (2022). Highly effective agro-waste based functional green adsorbents for toxic chromium (VI) ion removal from wastewater. J. Mol. Liq..

[60] Hossain M.T., Khandaker S., Bashar M.M., Islam A., Ahmed M., Akter R., Alsukaibi A.K.D., Hasan M.M., Alshammari H.M., Kuba T. (2022). Simultaneous toxic Cd (II) and Pb (II) encapsulation from contaminated water using Mg/Al-LDH composite materials. J. Mol. Liq..

[61] Perez J.P.H., Schiefler A.A., Rubio S.N., Reischer M., Overheu N.D., Benning L.G., Tobler D.J. (2021). Arsenic removal from natural groundwater using ‘green rust’: Solid phase stability and contaminant fate. J. Hazard. Mater..

[62] Agnel M.I., Grangeon S., Fauth F., Elkaim E., Claret F., Roulet M., Warmont F., Tournassat C. (2020). Mechanistic and Thermodynamic Insights into Anion Exchange by Green Rus. Environ. Sci. Technol..

[63] Khandaker S., Toyohara Y., Saha G.C., Awual M.R., Kuba T. (2020). Development of synthetic zeolites from bio-slag for cesium adsorption: Kinetic, isotherm and thermodynamic studies. J. Water Process Eng..

[64] Khandaker S., Toyohara Y., Kamida S., Kuba T. (2018). Effective removal of cesium from wastewater solutions using an innovative low-cost adsorbent developed from sewage sludge molten slag. J. Environ. Manage.

[65] Awual M.R., Yaita T., Kobayashi T., Shiwaku H., Suzuki S. (2020). Improving cesium removal to clean-up the contaminated water using modified conjugate material. J. Environ. Chem. Eng..

[66] Teo S.H., Ng C.H., Islam A., Abdulkareem-Alsultan G., Joseph C.G., Janaun J., Tau Y.H., Khandaker S., Islam G.J., Znad H. (2022). Sustainable toxic dyes removal with advanced materials for clean water production: A comprehensive review. J. Clean. Prod..

