# Supplementary material for: Selective Adsorption of Fluorine Contaminants from Spiked Wastewater via a Novel FeIII–CeIV-Based Layered Hydroxide Composite and Mechanism Analysis of Colloids and Surfaces
Source: Materials (Basel). 2025 Jun 5;18(11):2665. doi: 10.3390/ma18112665 (PMC12155687; doi:10.3390/ma18112665)
Supplement: Supplementary file 1 [file materials-18-02665-s001.zip › materials-3533199-supplementary.pdf]

Supporting information

Complementary specification

Chemicals and materials

The ferric sulfate ($Fe_3(SO_4)_2$, AR) and the ferrous sulfate heptahydrate ($FeSO_4$•$7H_2O$, AR) were both purchased online from the Haiwan reagent factory, China. Sulfuric acid (H_2SO_4, 98%) and sodium hydroxide pellets (NaOH, AR) were both purchased from Sinopharm chemical reagent Co. Ltd, Shanghai, China. The cerium sulfate ($Ce(SO_4)_2$, 99.9%, metal basis), sodium chloride (NaCl, 99.99%, metal basis), sodium nitrate ($NaNO_3$, AR), sodium nitrite ($NaNO_2$, AR), sodium bromide (NaBr, 99.99%, metal basis), potassium chloride (KCl, 99.99%, metal basis), and potassium fluoride (KF, GR) were all bought online from Aladdin, Shanghai, China.

Kinetic and isotherm models for the equilibrium adsorption experiments

Kinetic models

Four kinetic models including the pseudo-first-order model, the pseudo-second-order model, the intraparticle-diffusion model, and Elovich are given as Eqs. (S1) – (S4), respectively, where q_e (mmol·g^{-1}) and q_t (mmol·g^{-1}) are adsorption capacities at equilibrium and time t (min), respectively; k_1 and k_2 are pseudo-first-order (min^{-1}) and pseudo-second-order rate constants (g·mg^{-1}·min^{-1}), respectively; k_p is the intraparticle-diffusion rate constant (mmol·g^{-1}·min$^{-0.5}$); C indicates a boundary layer thickness, α is the initial adsorption rate constant of the Elovich model (mg·g^{-1} min^{-1}), and β is the desorption rate constant of the Elovich model (g·mg^{-1}).

$$\ln(q_e - q_t) = \ln q_e - k_1 t \quad \text{(S1)}$$

$$\frac{t}{q_t} = \frac{1}{k_2 q_e^2} + \frac{t}{q_e} \quad \text{(S2)}$$

$$q_t = k_p t^{0.5} + C \quad \text{(S3)}$$

$$q_t = \frac{1}{\beta}\ln(\alpha\beta) + \frac{1}{\beta}\ln(t) \quad \text{(S4)}$$

Breakthrough model in the column mode

The breakthrough model of Thomas is given as Eq. (S5), where, c_0 and c_t are the concentrations of F$^-$ ions in influent and effluent solutions, respectively; t is the operating time of the continuous flow in total (h); M is the mass of the adsorbent packed in the treatment container (g); Q is the flow rate (mL·min^{-1}) of the influent solution; k_{th} is the Thomas model rate constant.

$$\ln\left(\frac{c_0}{c_t} - 1\right) = \frac{k_{th} q_e M}{Q} - k_{th} c_0 t \quad \text{(S5)}$$

Tables

Table S1 Parameters and labels for the synthesis of DD-LHC

No.	Parameters			Labels
	Molar ratios of Fe^{III} to Ce^{IV}	Concentration of Fe^{III} (M)	Molar ratios of OH^- to Fe^{III}	
1	0.25	0.25	7:1	DD-LHC-1
2	0.5	0.25	7:1	DD-LHC-2
3	0.75	0.25	7:1	DD-LHC-3
4	1	0.25	7:1	DD-LHC-4
5	1.25	0.25	7:1	DD-LHC-5
6	0.75	0.5	7:1	DD-LHC-6
7	0.75	0.75	7:1	DD-LHC-7
8	0.75	0.5	8:1	DD-LHC-8
9	0.75	0.5	9:1	DD-LHC-9
10	0.75	0.5	10:1	DD-LHC-10
11	0.75	0.5	11:1	DD-LHC-11
12	0.75	0.5	12:1	DD-LHC-12

Table S2 Factors and levels in the CCD (α = 1.682)

Factors	Labels	Levels				
		Cubic point		Center point	Axial point	
		-1	1	0	-1.682	1.682
The initial concentration of influent (mM)	X_1	10	20	15	6.59	23.41
The initial pH	X_2	4	10	7	1.95	12.05
The flow rate (mL/min)	X_3	10	15	12.5	8.30	16.71

Table S3 Choices of ion electrodes and the requirements for the detection of F^-, Br^-, Cl^-, and NO_3^-

Target	Electrode model	Measuring range	pH condition	Reference electrode model	Calibration solution
F^-	pF-2-01	5×10^{-6} – 10^{-1}	--	232-01	KF
Br^-	PBr-1/1-01	5×10^{-6} – 10^{-1}	2 – 11	217	NaBr
Cl^-	PCl-1/1-01	5×10^{-6} – 10^{-1}	2 – 10	$C(K_2SO_4)$-1	KCl
NO_3^-	PNO3-1/1-01	10^{-5} – 10^{-1}	4 – 9	232	$NaNO_3$

Table S4 Significance analysis in the CCD layout (Response: V_{bre} (L))

Sources	df	*Adj.* SS [a]	*Adj.* MS [b]	F	P
Regression	9	10581.3	1175.70	228.03	0.000
Linear	3	9465.3	3155.11	611.95	0.000
X_1	1	2525.7	2525.73	489.88	0.000
X_2	1	6803.6	6803.62	1319.60	0.000
X_3	1	136.0	135.99	26.38	0.000
Square	3	838.3	279.42	54.19	0.000
X_1*X_1	1	7.8	7.80	1.51	0.247
X_2*X_2	1	825.4	825.42	160.09	0.000
X_3*X_3	1	0.5	0.50	0.10	0.763
Interaction	3	277.7	92.55	17.95	0.000
X_1*X_2	1	274.0	274.01	53.15	0.000
X_1*X_3	1	2.3	2.31	0.45	0.518
X_2*X_3	1	1.3	1.33	0.26	0.623
Error	10	51.6	5.16		
Lack-of-Fit	5	41.7	8.35	4.26	0.069
Pure Error	5	9.8	1.96		
Total	19	10632.8			

a: Adjusted sum of squares; b: Adjusted mean squares

R^2 = 99.25%, *Adj.* R^2 = 99.08%

Figures

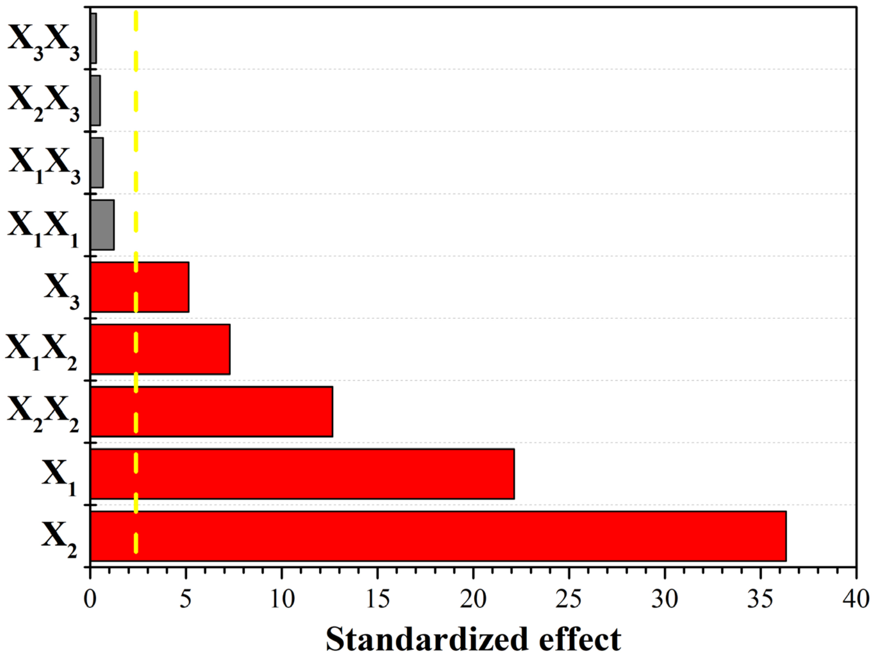

Fig. S1 Pareto chart of the standardized effects

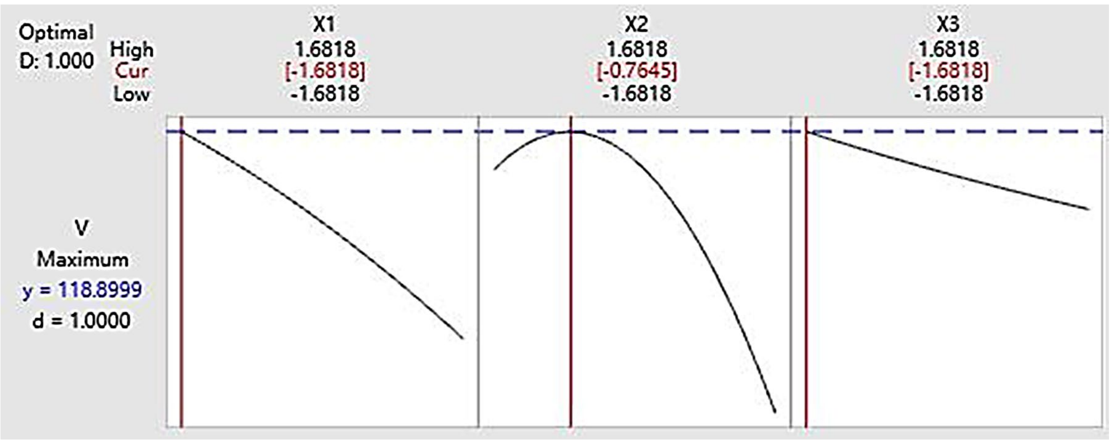

Fig. S2 Optimization of the CCD by the quadratic predictor and the desirability function
